# Foetal neural progenitors contribute to postnatal circuits formation ex vivo: an electrophysiological investigation

**DOI:** 10.1186/s13041-020-00619-z

**Published:** 2020-05-19

**Authors:** Matteo Manzati, Teresa Sorbo, Michele Giugliano, Laura Ballerini

**Affiliations:** 1 Neuron Physiology and Technology Lab, International School for Advanced Studies (SISSA), Neuroscience, Via Bonomea 265, I-34136 Trieste, Italy; 2 Neuronal Dynamics Lab, International School for Advanced Studies (SISSA), Neuroscience, Via Bonomea 265, I-34136 Trieste, Italy; 3 Department of Biomedical Sciences and Institute Born-Bunge, Universiteit Antwerpen, B-2610 Wilrijk, Belgium

**Keywords:** Microelectrode arrays, Cell electrophysiology, Neuronal networks, Neuronal progenitor cells, Hippocampus

## Abstract

Neuronal progenitor cells (NPC) play an essential role in homeostasis of the central nervous system (CNS). Considering their ability to differentiate into specific lineages, their manipulation and control could have a major therapeutic impact for those CNS injuries or degenerative diseases characterized by neuronal cell loss. In this work, we established an in vitro co-culture and tested the ability of foetal NPC (fNPC) to integrate among post-mitotic hippocampal neurons and contribute to the electrical activity of the resulting networks. We performed extracellular electrophysiological recordings of the activity of neuronal networks and compared the properties of spontaneous spiking in hippocampal control cultures (HCC), fNPC, and mixed circuitries ex vivo. We further employed patch-clamp intracellular recordings to examine single-cell excitability. We report of the capability of fNPC to mature when combined to hippocampal neurons, shaping the profile of network activity, a result suggestive of newly formed connectivity ex vivo.

## Introduction

Neuronal Replacement Therapy (NRT) directly addresses neurodegeneration by replacing dead cells with healthy new ones to restore compromised brain functions. Among NRT candidates, multipotent neural stem cells derived from embryonic tissue are the most promising. However, the mechanisms underlying the CNS functional improvements by NRT are not yet completely understood [1]. For this reason, we used a mixed population of foetal neural progenitor cells (fNPC) and cultured neurons dissociated from the rat hippocampus, as a new in vitro experimental model.

## Material and methods

### Cultures preparation

Primary HCC were obtained from Wistar rats at P2–3, as in [2]. fNPC were isolated from (E15) embryos, following [3]. pCCLsin.PPT.hPGK.EGFP.Wpre [4], a third-generation self-inactivating (SIN) lentiviral vector, was employed to express the enhanced Green Fluorescence Protein (eGFP) in fNPC, under the control of phosphoglycerate kinase (PGK) promoter. Generation and titration of the vector were as described [5] and transduction took place the same day of dissection. Mixed cultures were obtained by blending together fNPC and hippocampal neurons in 1:1 ratio in order to keep the same density as controls (fNPC and HCC). Thus, in all three conditions, cells were plated at a density of ⁓420 cells/mm^2^ on 0.01% poly-L-ornithine solution-coated glass coverslips (Orsatec GmbH, Bobingen, Germany) or at a density of ⁓3000 cells/mm^2^ on the inner area of MEAs, after coating with a 0.1% polyethylene-imine solution. Cover slips and MEAs were incubated at 37 °C, 5% CO_2_ for 15–17 DIV.

### Single-cell electrophysiology

Whole-cell patch-clamp was performed at 22 °C. Electrodes had a resistance of 5–8 MΩ when filled with a solution, containing (in mM) 120 K gluconate, 20 KCl, 10 HEPES, 10 EGTA, 2 MgCl_2_, 2 Na_2_ATP, pH 7.3. All recordings were performed under continuous perfusion (2 mL/min) of an extracellular solution containing (in mM) 150 NaCl, 4 KCl, 1 MgCl_2_, 2 CaCl_2_, 10 HEPES, 10 glucose, pH 7.4. Raw membrane potential was recorded in current clamp and the membrane passive properties (i.e. capacitance and input resistance) were estimated as in [6], holding the cells at − 56 mV. AP were evoked in current clamp upon delivering repeated depolarizing steps (1 nA, 5 ms, 15 times, 1 Hz), holding the cells at − 70 mV.

Pair recordings were performed by simultaneously patching two neurons, visualized in the same 40× microscopy field, alternatively holding one cell in current-clamp and the other in voltage-clamp mode. Brief current pulses were used to elicit an action potential in one cell, while monitoring the other (putative) postsynaptic cells for postsynaptic currents (PSCs).

### Network electrophysiology

Multisite extracellular recordings were carried out by regular TiN MEAs with 60 microelectrodes (60MEA200/30iR-Ti, MultiChannel Systems GmBH, Reutlingen, Germany) at 37 °C, in the presence of cell culture medium, and consisted of 1) 30 min, monitoring the spontaneous activity; 2) 30 min, monitoring the activity under GABA_A_ receptors blocker (bicuculline, BIC, 10 μM); 3) 5 min, validating the signal detection under tetrodotoxin (1 μM), a Na^+^ channels blocker.

### Immunofluorescence

Labelling with β tubulin III and DAPI was performed as described [7].

### Data analysis and statistics

**Fig. 1:**
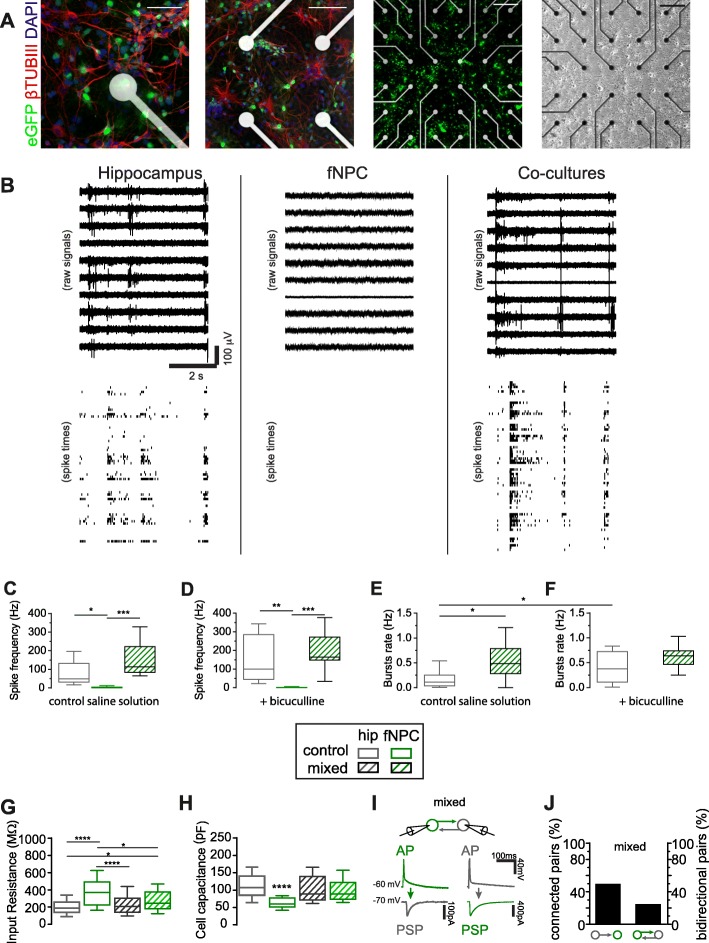
Extra- and intra-cellular recordings reveal heterogeneity of the electrophysiological phenotype, across culture conditions. **a** Representative mixed cultures (phase contrast and confocal microscopy, respectively left and right top images, scale bar = 200 μm); bottom images display β tubulin III^+^ neurons in red, eGFP^+^ neurons in green and cells nuclei in blue surrounding the MEA microelectrodes (scale bars = 100 and 50 μm). Sample raw extracellular electrical potentials detected at single sites of three distinct MEAs, representative of our experimental conditions (**b**). Results from all the experiments, quantified in **(c-f)**, suggesting that under both control conditions and disinhibition, HCC and hippocampal + fNPC mixed cultures, but not fNPC control cultures, detected spiking activity from the largest majority of MEA microelectrodes (not shown), with high rate of occurrence (**c**, **d**) and AP synchronization (**e, f**). **P < 0.01, ***P < 0.001, one-way ANOVA. When investigated by single-cell recordings, passive and active electrical cells’ properties reveal a distinct phenotype of fNPC in mixed cultures: input resistance (**g**) and capacitance (**h**). Note the inset explaining the legend. *P < 0.05, **P < 0.01, ***P < 0.001, ****P < 0.0001, Kruskal-Wallis. Pair recordings (**i-j**) were used to confirm the existence of synaptic connectivity between HCC and fNPC (10 cases out of 20) as well as the fraction of reciprocal connectivity (5 cases out of 20, see sample traces of panel **i**)

MEA data were analysed offline as in [8]: briefly, raw voltage traces were bandpass filtered (0.2–3 kHz) and AP extracted as peaks exceeding 5 times the signal median [9]. Bursts of synchronous AP were later identified as episodes of threshold crossing for the instantaneous product between the AP count and the active-electrodes count, binned at 25 ms [10]. Samples subjected to the same conditions were pooled together and their values expressed as mean ± SEM with n = number of cells or of MEAs. Statistical analysis was performed using GraphPad Prism 6 (GraphPad Software, San Diego, USA). Depending on whether assumptions were met for parametric testing, we used either one-way ANOVA followed by a Bonferroni-corrected multiple comparisons procedure or Kruskal Wallis analysis followed by Dunn’s multiple comparisons test. Significant differences were determined at *P* < 0.05. The convention to indicate the value of P is clearly indicated in the legend of Fig. 1. Numerical data were depicted (Fig. 1c-h) as box-and-whisker plots where the thick horizontal bar indicates the median value, the boxed area extends from the 25th to 75th percentiles while whiskers from the 10th to the 90th percentiles.

## Results

We compared three conditions: HCC (*n* = 8), fNPC (*n* = 8), and mixed cultures (n = 8). HCC and eGFP-tagged fNPC reorganized ex vivo into interconnected networks (Fig. 1). Bursts of AP, occurring synchronously in cells across the network were detected by distinct MEA microelectrodes (Fig. 1b**)**. These bursts emerge due to excitatory and inhibitory synaptic transmission interplay and intrinsic cell properties [11]. Pharmacological blockade of GABA_A_ receptors by BIC, altered network activity patterns (not shown). When cultured alone, fNPC displayed rare or no spiking activity, even under BIC.

The number of microelectrodes detecting repeated extracellular AP was then taken, for each MEA (*n* = 8 per condition), as a measure of synaptic and neuronal viability across the network (not shown). We observed a much higher number of active electrodes in mixed (45.0 ± 4.69) and HCC (38.5 ± 2.83) than in fNPC cultures (5.00 ± 1.95). Under BIC, the number of active electrodes was still higher in mixed (46.6 ± 3.26) and HCC (40.9 ± 5.27) than in fNPC cultures (5.63 ± 1.82). Next, we quantified the frequency of occurrence of spontaneous AP for every culture (Fig. 1c-d). We observed that the AP frequency differed, although not significantly, comparing HCC (76.7 ± 22.6 Hz) and mixed cultures (151 ± 32.2 Hz). Instead, the difference was significant both with HCC and mixed cultures, compared to fNPC cultures (2.66 ± 1.44 Hz).

Under BIC, the AP frequency in mixed cultures (190 ± 40.8 Hz;) was also higher, although not significantly, than in HCC (144 ± 44.4 Hz), and both were significantly different (Fig. 1d) than fNPC cultures (1.38 ± 0.565 Hz).

The emergence of AP episodic synchronization, a well-known correlate of synaptogenesis [12, 13], was studied by analysing the rate of occurrence of AP “bursts”, across each MEA (Fig. 1e). Interestingly, fNPC cultures never displayed bursting. Burst rate was significantly higher in mixed cultures (0.547 ± 0.131 Hz) than in HCC (0.163 ± 0.063 Hz). Burst lengths in mixed cultures (296.7 ± 67.3 ms) and HCC (186.3 ± 58.4 ms) did not differ significantly (*p* = 0.27). Under BIC, no bursting occurred in fNPC cultures (Fig. 1f), while a very similar degree of episodic synchronization was found in mixed (0.615 ± 0.094 Hz) and HCC (0.410 ± 0.109 Hz). Moreover, BIC had a significant effect on burst rate in HCC but not in mixed cultures.

We then performed single-cell intracellular recordings, comparing eGFP+ and eGFP- neurons across our conditions. The values of input resistance (397.7 ± 28.3 MΩ; *n* = 69) were significantly higher in fNPC compared to HCC (207.2 ± 14.8 MΩ; *n* = 56). Instead, fNPC in mixed cultured displayed no differences (283.6 ± 19.75 MΩ; *n* = 49) when compared to HCC (235.2 ± 23.7 MΩ; *n* = 37) (Fig. 1g). The membrane capacitance in fNPC (62.5 ± 2.3 pF; n = 69) displayed significative differences when compared to HCC (109 ± 4.7 pF; n = 56), and significant differences when compared to fNPC grown in mixed cultures (99.6 ± 4.9 pF; n = 49), as shown in Fig. 1h.

We then probed the active membrane properties upon injecting current pulses, while monitoring the membrane potential. All cell types responded with AP. However, despite similar peak amplitudes (average 100.8 ± 1.8 mV), AP rise time was significantly faster in fNPC (2.1 ± 0.1 ms; *n* = 63) than among all the other experimental conditions, while a significative different degree of maturation could be observed, comparing these values to fNPC in mixed cultures (2.6 ± 0.1 ms; *n* = 44). HCC responded with AP rise time of 2.8 ± 0.1 in all conditions.

Finally, we performed simultaneous pair recordings (*n* = 20) in mixed cultures to characterise the monosynaptic connectivity between fNPC and HCC (Fig. 1i-j). In 50% of the cases HCC neurons synapsed to fNPC and in 25% of the cases a reciprocal connection was found (Fig. 1i).

## Discussion

We evaluated whether fNPC integrate among and communicate with post-mitotic hippocampal neurons, by establishing a novel in vitro co-culture system and electrophysiological assay. Our recordings suggest the formation of functional hybrid networks differing from control cultures, resulting in qualitatively and quantitatively different emerging spontaneous AP synchronization.

Voltage-clamp intracellular recordings indicated a significant alteration of cell membrane passive properties, as fNPC grew in mixed cultures compared to control cultures, implying overall a maturation through a significant increase in cell size and number of ion channels of the cell membranes. Current-clamp experiments further suggest cellular maturation, as fNPC’s excitability was examined in response to a depolarizing stimulus. Pair recordings further supported our conclusions, by documenting the synaptic integration of fNPC in HCC microcircuits. Finally, it is tempting to speculate that the effect of BIC on the burst rate links fNPC differentiation to the excitatory/inhibitory balance of network activity, a possibility that is also supported by the lack of significant BIC effects on mixed cultures. This might be reinforced by the role of the reciprocal synaptic connectivity where fNPC are involved. Further studies, involving immunohistochemistry and an expansion of our paired-cell recordings performed here would still be needed to confirm these hypotheses.

## Data Availability

The datasets collected and analysed during the current study is available from the corresponding author on reasonable request.

## Abbreviations

NPC: Neuronal progenitor cells
fNPC: Foetal neuronal progenitor cells
HCC: Hippocampal control cultures
CNS: Central Nervous System
NRT: Neuronal Replacement Therapy
P2–3: Postnatal days 2–3
E15: Embryonic day 15
DIV: Days in vitro
AP: Action potentials
MEA: MicroElectrode Arrays
eGFP: enhanced Green Fluorescent Protein
PGK: Phosphoglycerate Kinase
TiN: Titanium Nitride
GABA: Gamma-Aminobutyric acid
BIC: Bicuculline

## References

[1] Grade S, Götz M (2017). Neuronal replacement therapy: previous achievements and challenges ahead. npj Regen. Med.

[2] Bosi S (2015). From 2D to 3D: novel nanostructured scaffolds to investigate signalling in reconstructed neuronal networks. Sci Rep.

[3] Aurand ER, Wagner JL, Shandas R, Bjugstad KB (2014). Hydrogel formulation determines cell fate of fetal and adult neural progenitor cells. Stem Cell Res.

[4] Follenzi A, Naldini L (2002). Generation of HIV-1 derived lentiviral vectors. Methods Enzymol.

[5] Brancaccio M, Pivetta C, Granzotto M, Filippis C, Mallamaci A (2010). Emx2 and Foxg1 inhibit gliogenesis and promote neuronogenesis. Stem Cells.

[6] Iansek R, Redman SJ (1973). An analysis of the cable properties of spinal motoneurones using a brief intracellular current pulse. J Physiol.

[7] Rauti R (2016). Graphene oxide Nanosheets reshape synaptic function in cultured brain networks. ACS Nano.

[8] Mahmud M, Pulizzi R, Vasilaki E, Giugliano M (2014). QSpike tools: a generic framework for parallel batch preprocessing of extracellular neuronal signals recorded by substrate microelectrode arrays. Front Neuroinform.

[9] Quiroga RQ, Nadasdy Z, Ben-Shaul Y (2004). Unsupervised spike detection and sorting with wavelets and superparamagnetic clustering. Neural Comp.

[10] van Pelt J, Wolters PS, Corner M, Michael A, Rutten WL, Ramakers GJA (2004). Long-term characterization of firing dynamics of spontaneous bursts in cultured neural networks. IEEE Trans Biomed Eng.

[11] Giugliano M, Darbon P, Arsiero M, Lüscher H-R, Streit J (2004). Single-neuron discharge properties and network activity in dissociated cultures of neocortex. J Neurophysiol.

[12] Marom S, Shahaf G (2002). Development, learning and memory in large random networks of cortical neurons: lessons beyond anatomy. Q Rev Biophys.

[13] Kamioka H, Maeda E, Jimbo Y, Robinson HPC, Kawana A (1996). Spontaneous periodic synchronized bursting during formation of mature patterns of connections in cortical cultures. Neurosci Lett.

